# Poor Accuracy of Methods Currently Used to Determine Umbilical Catheter Insertion Length

**DOI:** 10.1155/2010/873167

**Published:** 2010-05-10

**Authors:** Gerdina H. Verheij, Arjan B. te Pas, Ruben S. G. M. Witlox, Vivianne E. H. J. Smits-Wintjens, Frans J. Walther, Enrico Lopriore

**Affiliations:** Division of Neonatology, Department of Paediatrics, Leiden University Medical Centre, P.O. Box 9600, 2300 RC Leiden, The Netherlands

## Abstract

This study compares the methods of Dunn and Shukla in determining the appropriate insertion length of umbilical catheters. In July 2007, we changed our policy for umbilical catheter insertions from the method of Dunn to the method of Shukla. We report our percentage of inaccurate placement of umbilical-vein catheters (UVCs) and umbilical-artery catheters (UACs) before and after the change of policy. In the Dunn-group, 41% (28/69) of UVCs were placed directly in the correct position against 24% (20/84) in the Shukla-group. The position of the catheter-tip of UVCs in the Dunn-group and the Shukla-group was too high in 57% (39/69) and 75% (63/84) of neonates, respectively. UACs in the Dunn-group were placed directly in the correct position in 63% (24/38) compared to the 87% (39/45) of cases in Shukla-group. The position of the catheter-tip of UACs in the Dunn-group and the Shukla-group was too high in 34% (13/38) and 13% (6/45) of neonates, respectively. In conclusion, the Dunn-method is more accurate than the Shukla-method in predicting the insertion length for UVCs, whereas the Shukla-method is more accurate for UACs.

## 1. Introduction

Umbilical catheters are frequently required in the management of severely ill neonates. Umbilical-vein catheters (UVCs) can be used for intravenous administration of parenteral nutrition, hypertonic solutions, blood products, and medication. Umbilical-artery catheters (UACs) can be used for blood-sampling and continuous monitoring of blood pressure. However, the advantages of umbilical catheters must be carefully balanced against the potential risks. Several life-threatening complications have been associated with the use of umbilical catheters including catheter-related infections, intestinal necrosis, thrombosis, cardiac arrhythmias, myocardial perforation, and pleural and pericardial effusion [1–8]. Complications associated with umbilical catheterization may result from inappropriate positioning of the catheter. Accurate prediction of the insertion length of the catheter is therefore paramount, as well as the confirmation of the position after insertion by chest X-ray or with ultrasound [9, 10].

Several formulas and graphs using various body measurements have been proposed to predict the correct position of umbilical catheters [2, 11–16]. The two most widely used methods are Dunn [14] and Shukla and Ferrara [15]. The Dunn-method is based on the measurement of the shoulder-umbilicus length and uses nomograms to determine the insertion length of the catheters [14]. Dunn examined 50 neonates at necropsy. 19 were stillborn and the remaining 31 had died during the first week of life. Their birth weight ranged from 680 to 4027 gram. The Shukla-method uses equations based on birth weight [15]. Shukla retrospectively studied 43 neonates (mean birth weight 2037 [+/−1077] gram) with UACs and 10 (mean birth weight 2260 [+/−1144] gram) with UVCs. In a prospective study, Shukla inserted 25 UACs and 16 UVCs in 29 neonates using equations derived from the retrospective study. He found all catheter tips in acceptable positions. The accuracy of both methods to estimate the correct insertion length is not well known. Both methods have been developed based on a small group of neonates and have not been validated prospectively in larger groups of neonates.

We performed a study to compare the accuracy of both methods in determining the correct position of umbilical catheters.

## 2. Patients and Methods

In this prospective observational study, we compared the position of umbilical catheters before and after implementation of the Shukla-method at our department, in July 2007. In the first study period, from December 1st 2006 to June 1st 2007, the policy used to determine the insertion length of umbilical catheters was based on the Dunn-method. During this period two members of our group (GV and EL) observed that the Dunn-method was often associated with a too high position and significant inter- and intraobserver variation [17]. To avoid this variation-bias, the method of Shukla was implemented in our neonatal nursery and was used in the second study period from October 1st 2007 to April 1st 2008. During the two 6-month study periods, all neonates admitted to the Leiden University Medical Center who received umbilical catheters were included in the study. Parental consent was not necessary, because both the method of Dunn and Shukla are accepted methods to use for the positioning of umbilical catheters in the Netherlands. In accordance with Dutch legislation on retrospective observational studies, IRB approval was not required. Patients with fetal hydrops or major congenital abnormalities were excluded, because of possible interference with the calculated or measured insertion length of the catheters. We also excluded cases in which a UAC was mistakenly placed in the umbilical vein instead of an artery or a UVC was placed in an artery.

In the first study period, we measured the length from the tip of the neonate's shoulder to the umbilicus and used the nomograms of Dunn to determine the insertion length of the catheters. In the second study period, we calculated the insertion length with the equations of Shukla. To calculate the depth for inserting a UAC, we multiplied the neonate's weight in kilograms by 3 and added 9 cm. To calculate the depth for inserting a UVC we used the insertion length of the UAC, divided this number by 2, and added 1 cm [2, 14, 15].

We confirmed the depth of the catheter-tip using antero-posterior chest X-rays. The position was stated as the corresponding vertebra level. The primary outcome variable was the rate of UVCs and UACs ideally positioned on initial X-ray. Ideal position of the UVC was defined as the catheter-tip being visible between the 9th and 10th thoracic vertebrae on a chest X-ray [2, 18]. The position of the UVC was considered too high if the tip of the catheter was higher than the 9th thoracic vertebra, and too low if the tip was below the 10th thoracic vertebra. Ideal position of the UAC was defined as the catheter-tip being visible between the 6th and 10th thoracic vertebrae [11, 16, 18, 19]. The position of the UAC was considered too high if the tip of the catheter was higher than the 6th thoracic vertebra, and too low if the tip was below the 10th thoracic vertebra. Secondary outcomes were rates of too high and too low position and rate of complications. The following neonatal data were collected: success of insertion of catheters, gestational age at delivery, birth weight, gender, mortality, respiratory distress syndrome, mechanical ventilation, patent ductus arteriosus requiring medical treatment or surgical closure, necrotizing enterocolitis ≥ Bell stage II, culture-proven sepsis, and catheter-related complications such as thrombosis, cardiac arrhythmias, myocardial perforation, pleural and pericardial effusion. Ultrasound examination to rule out catheter-related complications (such as thrombosis) was not routinely performed in all neonates. Catheter-related complications were diagnosed only after clinical suspicion.

### 2.1. Statistical Analysis

Results of categorical variables were compared using Fisher's exact test or Chi-square test as appropriate. Student's *t*-test was used to compare normally distributed values between two groups. A *P*-value <.05 was considered to indicate a statistical significance. Analysis was performed using SPSS version 16 (SPSS, Inc., Chicago, Illinois, USA).

## 3. Results

During the study period, 220 patients received either a UVC or a UAC, or both. Four patients were excluded due to fetal hydrops (*n* = 2) and incorrect insertion of catheters (UVC in umbilical artery and UAC in umbilical vein) (*n* = 2). The remaining 216 patients were included in the study, 97 patients in the Dunn-group and 119 patients in the Shukla-group. All patients received a UVC and 144/216 patients also received a UAC.

Table 1 shows the baseline characteristics of both groups. Birth weight ranged from 575 to 6430 grams in the total group and gestational age from 25 to 42 weeks. Overall, UVCs were successfully introduced in 70% (153/220) of neonates and UACs were placed successfully in 58% (83/144). In the other neonates, the tip of the catheter could not be introduced in the vessel or (in UVCs) the tip was in the portal vein. If the tip of the UVC was in the portal vein, a second attempt to introduce a UVC next to the first UVC was made. This second attempt was successful in 23% (10/44) of cases. The vast majority of catheters (98%) were inserted by residents. We found no difference between the rate of successful and correct insertion between residents and experienced neonatologists.

**Figure 1:**
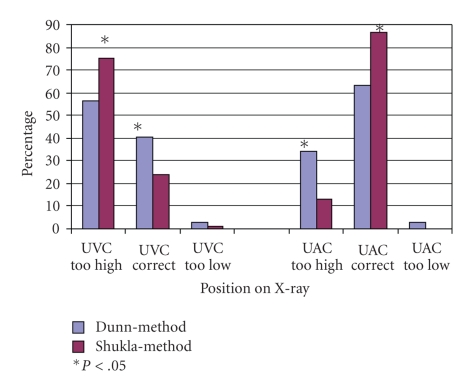
Positions of umbilical-artery catheters (UAC) and umbilical-vein catheters (UVC).

**Figure 2:**
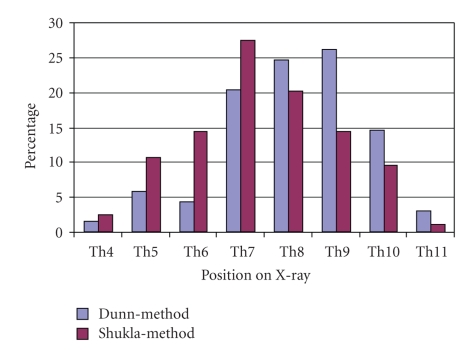
Positions of umbilical-vein catheters on X-ray.

**Figure 3:**
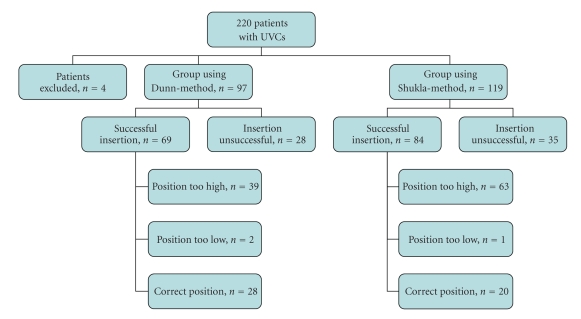
Flow-chart showing the position of the umbilical-vein catheters (UVCs) using the 2 different methods.

Figure 1 shows the percentages of UVCs and UACs placed correctly and incorrectly in the Dunn-group and Shukla-group. Positions of the tip of UVCs in the Dunn-group and Shukla-group ranged from the 4th to the 11th thoracic vertebrae on chest X-rays (Figure 2). In the Dunn-group, 41% (28/69) of UVCs were placed directly in the correct position against 24% (20/84) in the Shukla-group (*P* < .05). UVCs placed according to the Dunn-method (57%, 39/69) were less often in a too high position (above the 9th thoracic vertebra) compared to UVCs placed according to the Shukla-method (75%, 63/84) (*P* < .05). In the Dunn-group, 3% (2/69) of UVCs were placed too low and in the Shukla-group 1% (1/84) (*P* = .45). Catheter-tips of UVCs placed at two or more vertebrae higher than the 9th thoracic vertebra were found in 32% (22/69) in the Dunn-group and in 55% (46/84) in the Shukla-group (*P* < .01) (Figure 3).

**Figure 4:**
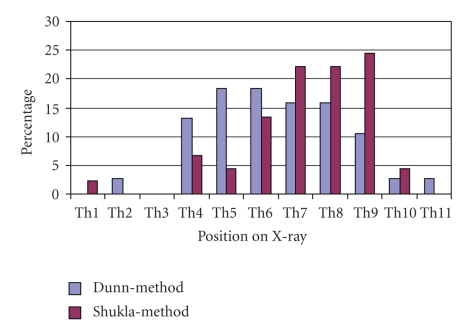
Positions of umbilical-artery catheters on X-ray.

**Figure 5:**
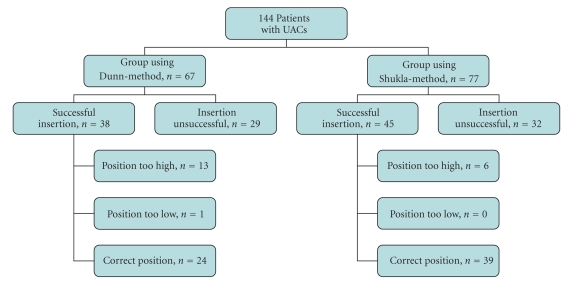
Flow-chart showing the position of the umbilical-artery catheters (UACs) using the 2 different methods.

Positions of the tip of UACs in the Dunn-group and Shukla-group ranged from the 1st to the 11th thoracic vertebrae on chest X-rays (Figure 4). UACs in the Dunn-group were placed directly in the correct position in 63% (24/38) compared to 87% (39/45) of cases in the Shukla-group (*P* < .05). The tip of UACs was placed too high in 34% (13/38) of cases in the Dunn-group and 13% (6/45) of cases in the Shukla-group (*P* < .05). One UAC in the Dunn-group was in a too low position (Figure 5).

One neonate in the Shukla-group developed a supraventricular tachycardia directly after umbilical catheterization. The chest X-ray showed malposition of the UVC-tip at the 6th thoracic vertebra. Catheter-related thrombosis, diagnosed by ultrasonography, occurred in 5/97 (5%) of patients in the Dunn-group and 3/119 (3%) of patients in the Shukla-group. The thrombi were located in the right atrium or inferior vena cava. Myocardial perforation, pericardial or pleural effusion did not occur during the study period.

## 4. Discussion

In this study, we assessed the accuracy of the two most commonly used methods to predict the appropriate insertion length of UVCs and UACs [2, 14, 15]. We observed that the overall accuracy of both methods is poor and both methods lead to too high positions of umbilical catheters. Interestingly, the Dunn-method is more accurate than the Shukla-method in predicting the appropriate insertion length for UVCs, whereas the Shukla-method is more accurate for the placement of UACs. High-positioned venous catheters (with the tip in the right atrium or deeper) are associated with cardiac arrhythmias, intracardiac thrombosis, pleural and pericardial effusions, and myocardial perforation and need to be avoided [3, 5–8, 20].

Thrombosis related to the catheter occurred in 8/216 (4%) in the total study group. Since ultrasonography to detect or rule out thrombosis was not routinely performed, data on the rate of catheter-related thrombi presented in this study should be interpreted with care as under-reporting cannot be ruled out.

The high rate of a too high position of UVCs observed in this study can be due to several reasons. Both Dunn and Shukla accepted in their original research a position of the UVC-tip in the right atrium [14, 15], but this position is now considered to be too high [21]. The optimal position for UVCs is at the junction of the inferior vena cava and the right atrium [9, 21]. This will correspond to the catheter-tip being visible between the 9th and 10th thoracic vertebrae on a chest X-ray, although positioning at the level of the 8th thoracic vertebra may also be adequate in some patients [9]. Also, both methods were developed based on a small sample size (*n* = 50 in the Dunn-study and *n* = 29 in the prospective part of the Shukla-study) and have not been validated prospectively in larger groups of neonates [14, 15]. In addition, the Dunn-method is limited by interobserver variation [17]. 

Our findings are in agreement with the results from a recent randomized controlled trial reported by Wright et al. [11], in which they report that placement of UACs using the Dunn-method leads to a too high position in many neonates. The aim of this trial was to establish the validity of a newly derived formula [(4 × birth weight) + 7 cm] compared to the Dunn-method. Wright et al. showed that the use of this formula significantly reduced the rate of UACs placed too high. In 3% (1/35) of neonates in which the new formula was used the UAC was too high, against 49% (19/39) in the control group in which the Dunn-method was used, (*P* < .0001). Whether this new formula is more accurate than the Shukla-method has not been studied and requires further investigation. Moreover, only very low-birth weight infants were included (<1500 gram), limiting the applicability of this new formula to this subgroup [11]. 

A major disadvantage of Wright's formula is the high rate (11%) of catheters positioned too low, which is of concern given the association with gut ischemia and thrombosis of renal and mesenteric arteries [21]. Ideally, UACs should either be placed in a high position (between the 6th and 10th thoracic vertebrae) or in a low position (between the 3rd and 5th lumbal vertebrae) [11, 19, 22, 23]. A recent Cochrane systematic review suggests that high positioning of UACs is associated with less ischemic complications [24]. 

Shukla and Ferrara verified their own formulas and found all catheter-tips in acceptable positions, but they only verified 26 UACs and 16 UVCs [15]. Weaver and Ahlgren based their equation to predict the insertion length of UACs on measurement of the heel-to-crown length of the neonate and reported that in 39 of 40 cases the catheter-tip was at the desired level [16]. Heel-to-crown length, however, is difficult to measure and this method is not widely used.

Although our study is limited by the non-randomized, nonblinded design, the catheters were inserted by almost 20 doctors and most of them were unaware of the advantages or disadvantages of both methods (in terms of accuracy). Another limitation is related to the confirmation of catheter position with X-ray. Whether radiography is the ideal diagnostic tool to determine the correct position of UVCs and UACs is controversial. Some authors advocate bedside real-time ultrasonography as the gold standard in verifying the position of umbilical catheters [9].

## 5. Conclusion

Although the method of Dunn is more accurate than the method of Shukla for determining the appropriate insertion length of UVCs, the Shukla-method is superior for determining the appropriate insertion length of UACs. However, it would not be practical to combine two different methods for the insertion of umbilical catheters, which may even lead to confusion and mistakes. Ideally, new and more reliable formulas should be developed for determining the correct insertion length of UVCs as well as UACs. These formulas should be validated in large studies of neonates including a wide range of gestational age and birth weight.

## Figures and Tables

**Table 1:** Baseline characteristics in Dunn-group versus Shukla-group.

	Dunn-group (*N* = 97)	Shukla-group (*N* = 119)	*P* *-*value
Male-*n* (%)	53 (55%)	68 (57%)	.78
Birth weight (grams)*	1997 ± 1223	1902 ± 988	.54
Gestational age at birth (weeks)*	32.3 ± 4.8	32.1 ± 4.6	.76
SGA-*n* (%)	10 (10%)	7 (6%)	.31
RDS-*n* (%)	31 (32%)	33 (28%)	.55
Mechanical ventilation-*n* (%)	57 (59%)	62 (52%)	.34
PDA-*n* (%)	18 (19%)	12 (10%)	.08
NEC-*n* (%)	3 (3%)	3 (3%)	1.0
Sepsis-*n* (%)	25 (26%)	24 (20%)	.33
Catheter-related thrombosis-*n* (%)	5 (5%)	3 (3%)	.47
Cardiac arrhythmia-*n* (%)	0 (0%)	1 (1%)	1.0
Mortality-*n* (%)	7 (7%)	9 (8%)	1.0
Successful UVC insertion-*n* (%)	69 (71%)	84 (71%)	1.0
Successful UVC insertion at first attempt-*n*/*N* (%)	63/97 (65%)	80/119 (67%)	.77
Successful UVC insertion at second attempt-*n*/*N* (%)	6/19 (32%)	4/25 (16%)	.46
Successful UAC insertion-*n*/*N* (%)	38/67(57%)	45/77(58%)	.78

SGA: small for gestational age; RDS: respiratory distress syndrome; PDA: persistent ductus arteriosus; NEC: necrotizing enterocolitis; UVC: umbilical-vein catheter; UAC: umbilical-artery catheter.

*Values are given as mean ± SD.
